# Improved malaria case management in formal private sector through public private partnership in Ethiopia: retrospective descriptive study

**DOI:** 10.1186/s12936-016-1402-7

**Published:** 2016-07-11

**Authors:** Mesele D. Argaw, Asfawesen GY. Woldegiorgis, Derebe T. Abate, Mesfin E. Abebe

**Affiliations:** Private Health Sector Project, Abt Associates Inc. In Ethiopia, P. O. Box 2372, 1250 Addis Ababa, Ethiopia

**Keywords:** Malaria, Case management, Public private partnership, Formal private sector

## Abstract

**Background:**

Malaria is a major public health problem and still reported among the 10 top causes of morbidity and mortality in Ethiopia. More than one-third of the people sought treatment from the private health sector. Evaluating adherences of health care providers to standards are paramount importance to determine the quality and the effectiveness of service delivery. Therefore, the aim of this study was to evaluate the contribution of public private mix (PPM) approach in improving quality of malaria case management among formal private providers.

**Methods:**

A retrospective data analysis was conducted using 2959 facility-months data collected from 110 PPM for malaria care facilities located in Amhara, Dire Dawa, Hareri, Oromia, Southern Nation Nationalities and Peoples and Tigray regions. Data abstraction formats were used to collect and collate the data on quarterly bases. The data were manually cleaned and analysed using Microsoft Office Excel 2010. To claim statistical significance non-parametric McNemar test was done and decision accepted at P < 0.05.

**Results:**

From April 2012–September 2015, a total of 873,707 malaria suspected patients were identified, of which one-fourth (25.6 %) were treated as malaria cases. Among malaria suspected cases the proportion of malaria investigation improved from recorded in first quarter 87.7–100.0 % in last quarter (X^2^ = 66.84, P < 0.001). The majority (96.0 %) were parasitologically-confirmed cases either by using microscopy or rapid diagnostic tests. The overall slid positivity rate was 25.1 % of which half (50.7 %) were positive for *Plasmodium falciparum* and slightly lower than half (45.2 %) for *Plasmodium vivax*; the remaining 8790 (4.1 %) showed mixed infections of *P. falciparum* and *P. vivax*. Adherence to appropriate treatment using artemether-lumefantrine (AL) was improved from 47.8 % in the first quarter to 95.7 % in the last quarter (X^2^ = 12.89, P < 0.001). Similarly, proper patient management using chloroquine (CQ) was improved from 44.1 % in the first quarter to 98.12 % in the last quarter (X^2^ = 11.62, P < 0.001).

**Conclusions:**

This study documented the chronological changes of adherence of health care providers with the national recommended standards to treat malaria. The PPM for malaria care services significantly improved the malaria case management practice of health care providers at the formal private health facilities. Therefore, regional health bureaus and partners shall closely work to scale up the initiated PPM for malaria care service.

## Background

Malaria is caused by the protozoan parasite of the genus *Plasmodium* and transmitted by *Anopheles* mosquitoes. Globally, it is an important public health problem. According to the World Health Organization (WHO) global malaria report 2015, there were an estimated 214 million in 2015 (range 194–303 million) cases. Most of the estimated cases (88 %) occurred in WHO African Region. In the same year, an estimated 438,000 deaths were reported, mostly (90 %) in the African Region [1].

In Ethiopia, malaria is a major public health problem. Approximately over 50 million (60 %) of the population live in malaria endemic area, mainly at altitudes below 2000 m above sea level [2]. According to Ethiopian Federal Ministry of Health (EFMOH 2013/2014), there were 57,503 public sector malaria hospitalizations, 4.9 million malaria outpatient cases, and the majority 2.9 million were laboratory-confirmed *Plasmodium falciparum* outpatient malaria cases, and 1.2 million were *Plasmodium vivax* cases [3].

Malaria is a significant impediment to social and economic development in Ethiopia. In endemic areas, malaria has affected the population during planting and harvesting seasons, cutting down productive capacity at a time when there is the greatest need for agricultural work. The disease has also been associated with loss of earnings, low school attendance, and high treatment cost [3–5].

In the last 5 decades, Ethiopia has executed all three WHO recommended malaria prevention and control strategies i.e. early diagnosis and prompt treatment, vector control and epidemic prevention and control [4, 6]. Currently, malaria care services in public health facilities are offered free of charge at all three levels of the health care tier system. Despite the effort made by the government to improve access and quality of services in public health facilities, significant numbers of the community members sought treatment from the private health sector [7–9].

On one hand, evidences from countries with different modality working with private health sector on malaria case management revealed improved quality of services [10–14]. On the other hand, there are reported challenges facing the health system due to unregulated private sector through poor adherence to the nationally recommended standards for malaria case management [15, 16]. In the context of malaria elimination, working with the private health sector is essential to ensure complete and timely reporting of all malaria cases and ensuring access to effective case management for people seeking treatment from private providers [1]. WHO developed the Global technical strategy for malaria 2016–2030, which sets the most ambitious targets for reduction in malaria cases and deaths since the malaria eradication era began [17]. This strategy was developed in line with the roll back malaria (RBM) partnership’s Action and investment to defeat malaria, to ensure shard goals and complementarity. The strategy has three main building blocks. The first pillar is to ensure universal access to malaria prevention, diagnosis and treatment. The second pillar is to accelerate efforts towards elimination of malaria and the third pillar is to transform malaria surveillance into a core intervention [17, 18].

In many developing countries the private health sector provides public health care and services for about one half of their population [1, 19, 20]. The situation in Ethiopia is quite similar with other SSA countries [19]. However, very few studies were documented on the role of private health facilities on malaria control and the quality of care in Ethiopia [21–23]. Jerne et al. state that out of 102 survey facilities in Oromia Regional State of Ethiopia, 86.0 % were providing malaria diagnosis and treatment services [21]. They also stated that the private health sector were not part of malaria case management training and didn’t get opportunity to be familiar with the most resent recommendations [21, 22]. Moreover, there was no strong established system to ensure the efficacy of drug accessed through private sector [17]. On top of these, the cost of full dose of artemether-lumefantrine (Coartem^®^) available through the private sector was found to be high and challenges the affordability of services to the general population.

Public Private Partnership (PPP) for malaria care service in Ethiopia has been implemented by six Regional State Health Bureaus and United State Agency for International Development (USAID) Funded Private Health Sector Programme (PHSP) (2009–2015). PHSP provided technical support for Regional Health Bureaus to take the leadership and stewardship on PPP and private facilities to be committed for the success of the national vision ‘seeing malaria free Ethiopia’. One hundred ten private health facilities engaged in the implementation of the malaria care services through initiated partnerships [19, 24, 25].

This retrospective study was conducted to analyse forty-two months’ health facility quarterly reports on malaria service delivery to assesses magnitude of cases and adherence of health care workers on the national standards. The result of this study will be useful for policy-makers, programme managers and health care workers for evidence based decision for quality service delivery.

## Methods

### Study area

Ethiopia is located in the horn of Africa with an area of estimated 1.1 million sq. km [26]. This data analysis covers five regional state and one city administration where over 54.5 million people live at risk of malaria [3].

### Process of establishing PPP for malaria care

Private Health Sector Programme (PHSP) was a 6 years project (September 2009–September 2015), funded by United States Agency for International Development (USAID). PHSP was the successor of Private Sector Project (PSP), which has piloted Public Private Mix Directly Observed Therapy Short Course (PPM_DOTS) and Human Immuno-deficiency Virus (HIV) programs in Ethiopia and concluded with recommendation to scale up the approach to maximize the health impact of the partnership [19, 24, 25].

**Fig. 1:**
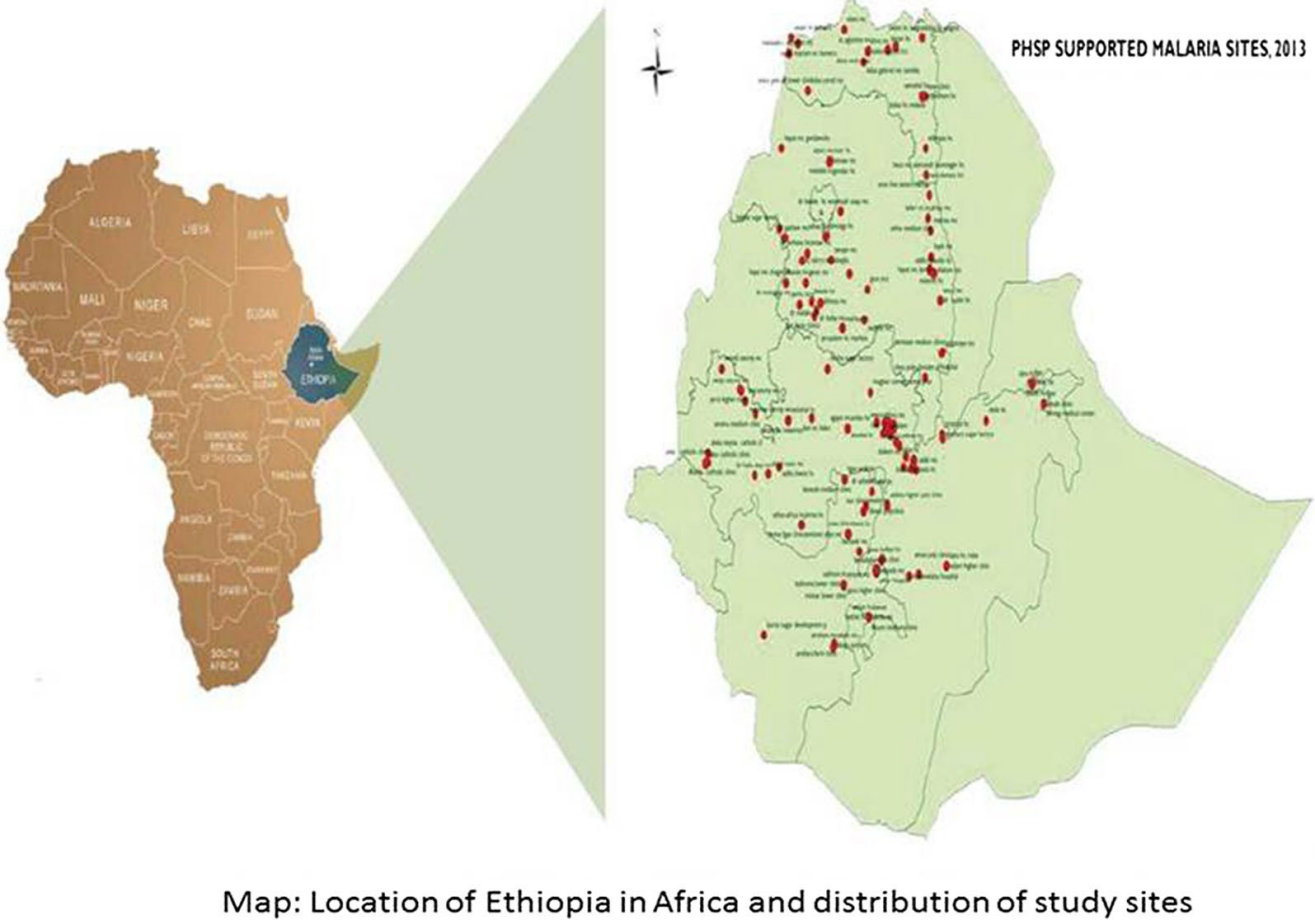
Map of location of Ethiopia in Africa and distribution of PPP for malaria care facilities. Map of study area with distribution of Public Private partnership for malaria care health facilities in Ethiopia

PHSP provided its technical support in the implementation of PPM for human immuno-deficiency virus (HIV) acquired immune deficiency syndrome (AIDS), tuberculosis (TB), malaria, family planning (FP), sexually transmitted infections (STI) programmes for five regional states and two city administrations namely: Amhara, Oromia, Tigray, Southern Nations Nationalities and Peoples (SNNP), Hareri Regions and Dire Dawa and Addis Ababa City Administration. Moreover, PHSP built the capacity of 342 private health facilities, primarily private for profit, followed by private not for the profit (faith based organization) and the third group were work place facilities; with the goal of establishing effective public private partnership for improving access to and demand for quality public health services with affordable costs. The malaria programme was implemented in 110 private health facilities (Fig. 1) [24].

### Foundation

**Fig. 2:**
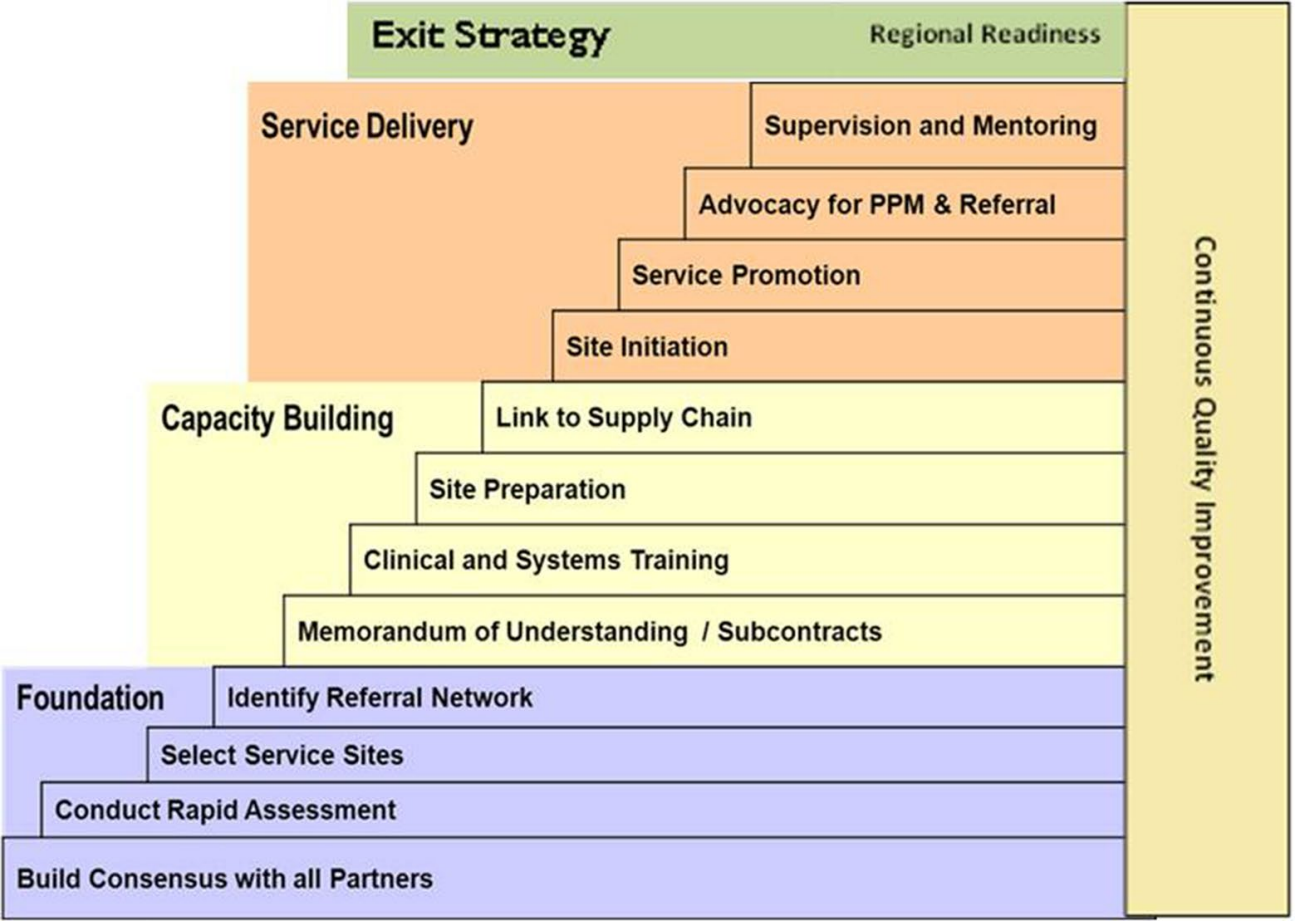
Private health sector programme implementation strategies. Figure depicting the step ladder fashion implementation strategies followed by the project which includes foundation, capacity building, service delivery, exit and continuous quality improvement cycles

PHSP has implemented its project using its programme implementation strategies [24] with step ladder fashion (Fig. 2). The first phase of the implementation strategy is dedicated to construct the foundation of PPP approaches. PHSP has conducted preliminary discussions with all Regional State Health Bureaus (RHBs). Then, consensus building workshops were held with delegates of public sector, private sector and other relevant stakeholders. PHSP in collaboration with RSHBs conducted facility readiness assessment from January through September 2012. Using a predetermined objective criteria like service integration, malaria case load, human resources, willingness and commitment of private health facilities owners, 110 health facilities i.e. seven Primary (Lower Clinics), 10 Hospitals, 37 Higher Clinics and 56 Medium Clinics were selected [24]. Moreover, Referral directory were developed and distributed to all actors for smooth networking. Therefore, this was the time which builds the capacity of public sector leadership and governance in owning the partnerships at regional health bureaus and its line structures.

### Capacity building

Before commencing the PPP for malaria care services, PHSP provided a team based trainings for case, laboratory and supply chain managers. The staff underwent 4 days of malaria case management and malaria diagnosis methods trainings. The third person attended nationally recommended 3 day training on supply chain management using standard operating procedure (SOP) for integrated pharmaceutical logistics system (IPLS).

In the implementation strategy after working on foundation, capacity building would resume in the parameter of clinical and non-clinical areas which include: training for private health facility owners on business management, signing of memorandum of understandings, linking facilities to public health supplies system and site preparation. During the study periods 344 malaria case managers were trained in twelve sessions. One hundred eighty five laboratory personnel were trained on malaria diagnosis, internal quality control (IQC), and external quality assurance (EQA) furthermore 140 supply chain mangers were trained in five sessions.

### Service delivery

The third and final steps are service delivery which includes service initiation, advocacy, demand creation supervision and mentoring. On a quarterly basis, technical assistances for all facilities was provided by a team of malaria expert from Woreda health office, laboratory quality officer, pharmacy mentor and programme officers. In addition, demands were created using 347 spot health radio messages in five local languages i.e. Amharic, Hareri, Oromiffa, Somali and Tigrigna, distribution of 168,500 patient brochures and 29,000 posters [24].

### Quality assurance

PHSP adopt, print and distribute a set of malaria morbidity and mortality register, comprehensive laboratory register, weekly reporting forms, national malaria guidelines and job aids. Furthermore, joint supportive supervisions were conducted on quarterly bases by a team of malaria experts from public sector, clinical officer, laboratory quality officer and pharmacy mentor.

The established partnerships need commitment of private health facilities to serve the community only with consultation and laboratory service fees. As per the signed Memorandum of Understanding (MOU) with or between RHB, confirmed *P. falciparum* cases should get AL (Coartem) for free of charge while *P. vivax* cases should be treated with chloroquine. In addition, the health facilities are expected to document the result of IQC and EQA results. Finally, the overall implementation of malaria case detection and management is verified through continuous quality improvement approaches [24].

### Data collection methods and data quality

This retrospective descriptive study [27] was conducted to determine malaria prevalence and adherence of health care providers to national standards using forty-two months or 2959 facility-months data i.e. from April 2012–September 2015. The data were collected from 110 Public Private Partnership (PPP) for malaria care facilities located in six regional states of Ethiopia. Data were collected using the pretested data abstraction form through reviewing primary source from comprehensive laboratory and malaria morbidity registers which consists of age, sex, date seen at health facility, diagnosis, treatment, history of admission, referral and outcome of admitted malaria patients. The tool has facility identifiers, data collection period and detail malaria case information.

In all PHSP supported private health facilities, malaria was diagnosed using standard operating procedure either using Giemsa (3 or 10 %) stained blood film or multi species malaria rapid diagnostic test kits (RDT). Only primary clinics (lower clinics) were expected to use RDTs to diagnose malaria. The data were collected by nine team composed of trained twenty four public health professionals (regional programme coordinators and program officers) and the data quality were ensured through regularly conducted data quality assessment by continuous quality improvement experts. The teams found margins of errors of less than 3 % [20].

### Data analysis

The summaries of quarterly reports were transferred to continuous quality improvement team through Open Data Kit (ODK) using smart phones. For statistical analysis the data were exported to Microsoft Excel 2010. The data were cleaned and checked for consistencies. Descriptive statistical analysis [27] (Frequency distribution and line graphs for trend analysis) were made. Botma et al. [28] recommended a non-parametric statistical analysis, McNemar Chi square test for paired or dependent proportions. For this retrospective descriptive study, McNemar’s test is selected, where each nominal data in the first quarter was paired with the last quarter data. Statistically significant relationship was claimed at P < 0.05 [28].

### Ethical clearance

The research protocol of this retrospective study was not reviewed by research ethics committee. As one of the project activity permission to use the data were sought and obtained from Private Health Sector Project, Abt Associated Inc. in Ethiopia. Patient identifier information was not collected. As per the requirement of the public health system summarized information’s were submitted to six Regional Health Bureaus (RHBs) on quarterly and annual bases.

## Results

### Descriptive information

A complete set of 2959 months-facility malaria morbidity data were collected on quarterly bases from 110 malaria care services facilities located in six regional states of Ethiopia. Between the initiation of PPP for malaria care services and September 2015, a total of 873,707 malaria suspected patients were identified, of which 223,293 (25.6 %) were treated as malaria cases. Almost all 214,259 (96.0 %) were parasitological confirmed either using microscopy or malaria RDTs. The rest 9034 (4.0 %) were diagnosed by clinical signs or symptoms as presumed malaria cases (Table 1).

**Table 1:** Malaria suspected, parasitological confirmed and clinically identified malaria cases in Ethiopia, April 2012–September 2015

Years	Malaria suspected cases	Investigated for malaria	%	Malaria cases (confirmed + clinical)^a^	%	Confirmed malaria cases	%	Clinical malaria cases	%
2012 (1)	71,800	62,455	87.0	26,817	37.3	24,698	92.1	2119	7.9
2013 (2)	292,986	288,225	98.4	89,985	30.7	84,080	93.4	5905	6.6
2014 (3)	336,250	328,760	97.8	74,566	22.2	73,673	98.8	893	1.2
2015 (4)	172,671	172,554	99.9	31,925	18.5	31,808	99.6	117	0.4
Grand total	873,707	851,994	97.5	223,293	25.6	214,259	96.0	9034	4.0

^a^χ^2^ = 14.061, df = 3, χ^2^/df = 4.69, P (χ^2^ >14.061) = 0.0028

The majority (63.7 %) of malaria suspected cases were served at medium clinics, followed by higher clinics (18.7 %). The third largest group of patients (13.8 %) was served in lower clinics and the rest of malaria suspected cases (3.8 %) were served in Hospitals.

**Fig. 3:**
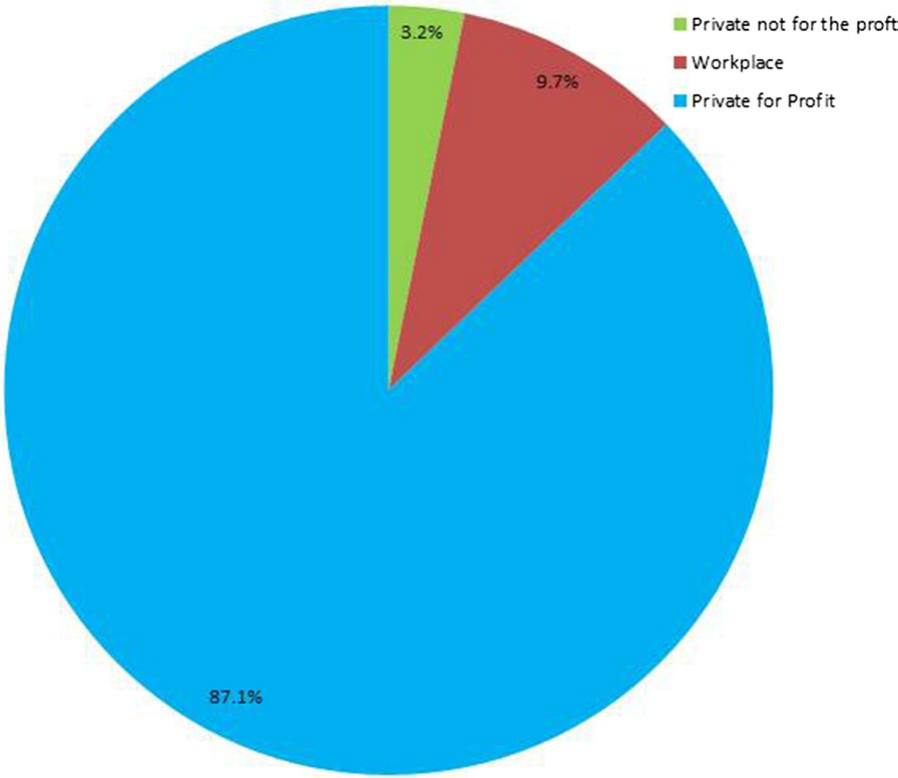
Pie chart depicted proportion of malaria care services beneficiaries by ownership of facilities, April 2012–September 2015 (n = 223, 293)

The majority 133,876 (60.0 %) of malaria patients were males. However, this gender difference in utilization of the service among malaria patient increased when the age group increased from lower to next higher age category. Two-third (68.9 %) were patients in the age category 15 years old or more, followed by 15.8 % were children 5–14 years old and the rest 15.2 % were under 5 years old children (Table 2). The majority 87.1 % of malaria suspected cases was serviced in private for profit facilities, followed by 9.7 % of malaria suspected patients were served in workplace facilities (Fig. 3).

**Table 2:** Distribution of malaria by age, sex and pregnancy status in Ethiopia, April 2012–September 2015

Year	0–4 years		5–14 years		15 + years		Males		Females	
	M	F	M	F	M	F	Freq.	%	Freq.	%
2012 (1)	2495	1813	2361	1862	10,490	7796	15,346	57.2	11,471	42.8
2013 (2)	8207	6282	8910	6434	35,987	24,165	53,104	59.0	36,881	41.0
2014 (3)	6145	4685	6260	4832	32,830	19,814	45,235	60.6	29,331	39.3
2015 (4)	2296	1708	2468	1918	15,427	8108	20,191	63.2	11,734	36.7
Grand total	19,143	14,488	19,999	15,046	94,734	59,883	133,876	60.0	89,417	40.0

### Malaria diagnosis

**Fig. 4:**
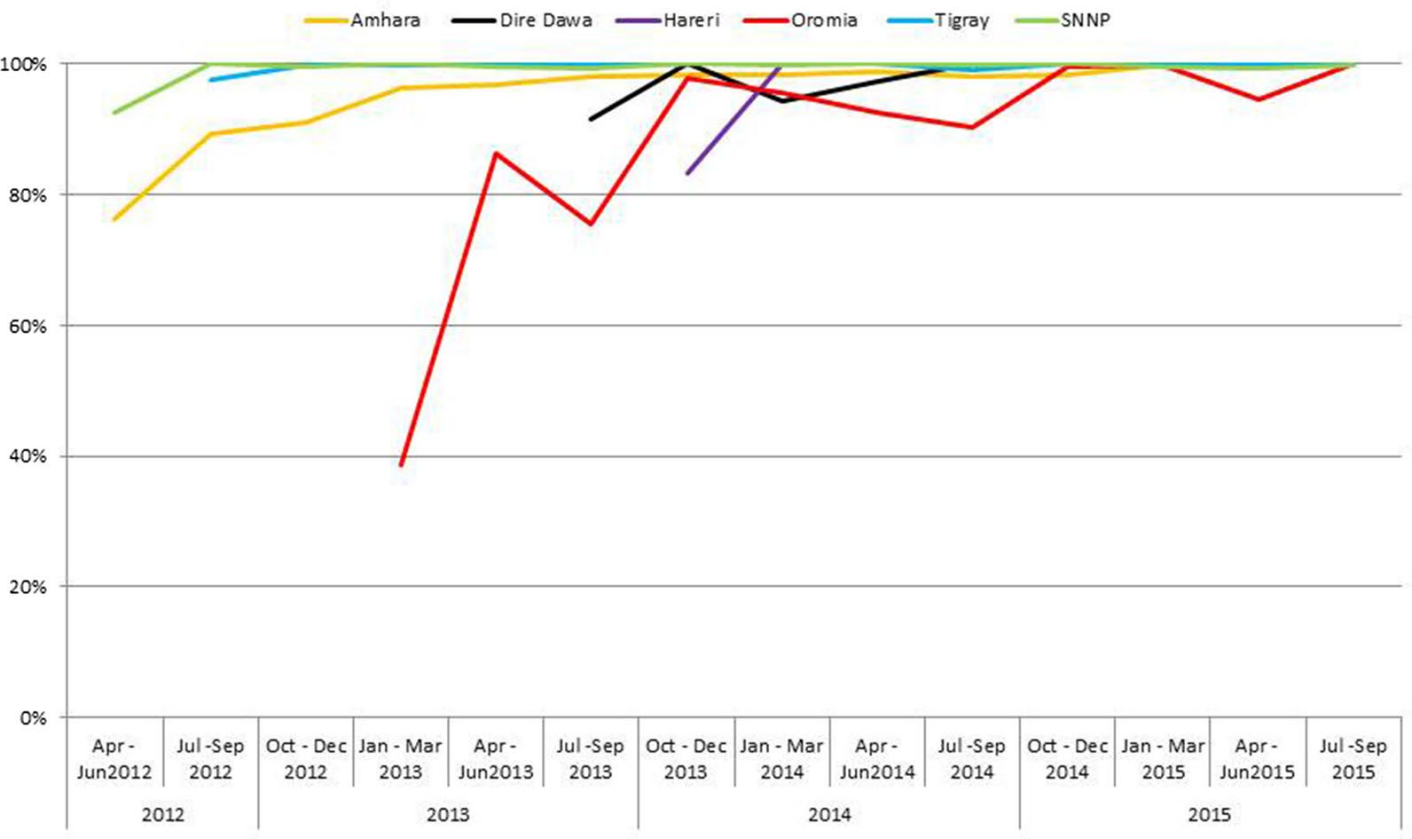
*Line chart* showing the proportion of confirmed malaria cases treated by region, 2012–2015

The malaria microscopy slide positivity rate was 24.5 % (198,066/807,275). Almost half of confirmed cases (50.4 %) were *P. falciparum*, 45.6 % were *P. vivax* (and the rest (4.1 %) were mixed species *P. falciparum*/*P. vivax* infections (Table 3). The malaria RDT positivity rate was 36.2 %. The Plasmodium species identified using RDT: 55.0 % were *P. falciparum*, 40.2 % were *P. vivax,* and 4.7 % were mixed infections (Table 4). The overall malaria parasite detection rate (either using microscopy or RDT) was 25.1 % (214,259/851,994). And the proportion of Plasmodium species confirmed in PPP facilities consists of 50.7 % were *P. falciparum*, 45.2 % were *P. vivax* and 4.1 % were mixed infections (Table 3). Making malaria diagnosis according to the national standards with parasitological confirmation was significantly improved from 87.7 % during the first 3 months to almost 100.0 % in the last 3 months, and PPP for malaria care facilities showed up their commitment with sustaining the results (Fig. 4).

**Table 3:** Malaria parasite detection rates using either Microscopy or RDT in PPP facilities Ethiopia, April 2012–September 2015

Year	Test type	Test done	Positive	Prevalence %	Pf	Pf %	Pv	Pv %	Mixed Pf/Pv	Mixed %
2012 (1)	BF	60,727	23,925	39.4	12,825	53.6	9503	39.7	1597	6.7
	RDT	1728	773	44.7	415	53.7	291	37.6	67	8.7
	BF and RDT	*62,455*	*24,698*	*39.5*	*13,240*	*53.6*	*9794*	*39.7*	*1664*	*6.7*
2013 (2)	BF	271,680	77,404	28.5	38,936	50.3	34,362	44.4	4106	5.3
	RDT	16,545	6676	40.4	3955	59.2	2558	38.3	163	2.4
	BF and RDT	*288,225*	*84,080*	*29.2*	*42,891*	*51.0*	*36,920*	*43.9*	*4269*	*5.1*
2014 (3)	BF	307,573	66,407	21.6	34,264	51.6	30,385	45.8	1758	2.6
	RDT	21,187	7266	34.3	4185	57.6	2673	36.8	408	5.6
	BF and RDT	*328,760*	*73,673*	*22.4*	*38,449*	*52.2*	*33,058*	*44.9*	*2166*	*2.9*
2015 (4)	BF	167,295	30,330	18.1	13,768	45.4	15,998	52.7	564	1.9
	RDT	5259	1478	28.1	356	24.1	995	67.3	127	8.6
	BF and RDT	*172,554*	*31,808*	*18.4*	*14,124*	*44.4*	*16,993*	*53.4*	*691*	*2.2*
Sub total BF		807,275	198,066	24.5	99,793	50.4	90,248	45.6	8025	4.1
Sub total RDT		44,719	16,193	36.2	8911	55.0	6517	40.2	765	4.7
Grand total		851,994	214,259	25.1	108,704	50.7	96,765	45.2	8790	4.1

Annual summary are presented with italics font

**Table 4:** Number of regions, health facilities, adherence to laboratory investigation and recommended treatment (April 2012–September 2015)

Time/quarter	Number of active regions	Malaria lab test done	%	Positive laboratory test	%	Appropriate AL (Coarterm)	%	Appropriate CQ (Chloroquine)	%
	Private health facilities	Malaria suspected		Malaria lab test done		AL illegible		CQ illegible	
Apr–Jun 2012	2	17,984	87.77	7220	40.15	2501	47.88	1425	44.19
	39	20,489		17,984		5223		3225	
Jul–Sep 2012	3	44,471	86.67	17,478	39.30	9648	81.76	6569	100.00
	57	51,311		44,471		11,800		6569	
Oct–Dec 2012	3	67,511	96.67	24,569	36.39	10,750	68.22	7293	76.36
	57	69,834		67,511		15,758		9551	
Jan–Mar 2013	4	69,091	98.61	18,037	26.11	6982	56.08	6483	70.40
	77	70,062		69,091		12,449		9209	
Apr–Jun 2013	4	78,297	99.61	20,216	25.82	9825	86.25	6706	71.01
	78	78,606		78,297		11,391		9444	
Jul–Sep 2013	5	73,326	98.44	21,258	28.99	10,076	74.82	5727	65.71
	88	74,485		73,326		13,467		8716	
Oct–Dec 2013	6	96,721	93.40	27,901	28.85	14,373	85.65	9507	83.81
	100	103,551		96,721		16,781		11,344	
Jan–Mar 2014	6	85,498	99.83	17,357	20.30	8897	99.02	8421	98.84
	110	85,645		85,498		8985		8520	
Apr–Jun 2014	6	72,869	99.79	12,552	17.23	6108	97.43	6320	98.11
	110	73,019		72,869		6269		6442	
Jul–Sep 2014	6	73,673	99.51	15,863	21.53	9082	95.87	6699	99.22
	99	74,035		73,673		9473		6752	
Oct–Dec 2014	4	51,875	99.91	12,255	23.62	5402	81.99	5354	93.68
	41	51,924		51,875		6589		5715	
Jan–Mar 2015	4	42,766	99.96	6526	15.26	2167	98.10	4264	98.41
	43	42,782		42,766		2209		4333	
Apr–Jun 2015	4	37,546	99.86	6103	16.25	2442	95.13	3587	100.00
	43	37,597		37,546		2567		3587	
Jul–Sep 2015	4	40,367	100.00	6924	17.15	3417	95.79	3295	98.12
	43	40,368		40,367		3567		3358	
Total		851,995	97.51	214,259	25.15	101,670	80.35	81,735	84.47
		873,708		851,995		126,528		96,765	
	McNemar’s test	66.84	P < 0.001	26.67	P < 0.001	12.89	P < 0.001	11.62	P < 0.001

### Malaria case management

**Fig. 5:**
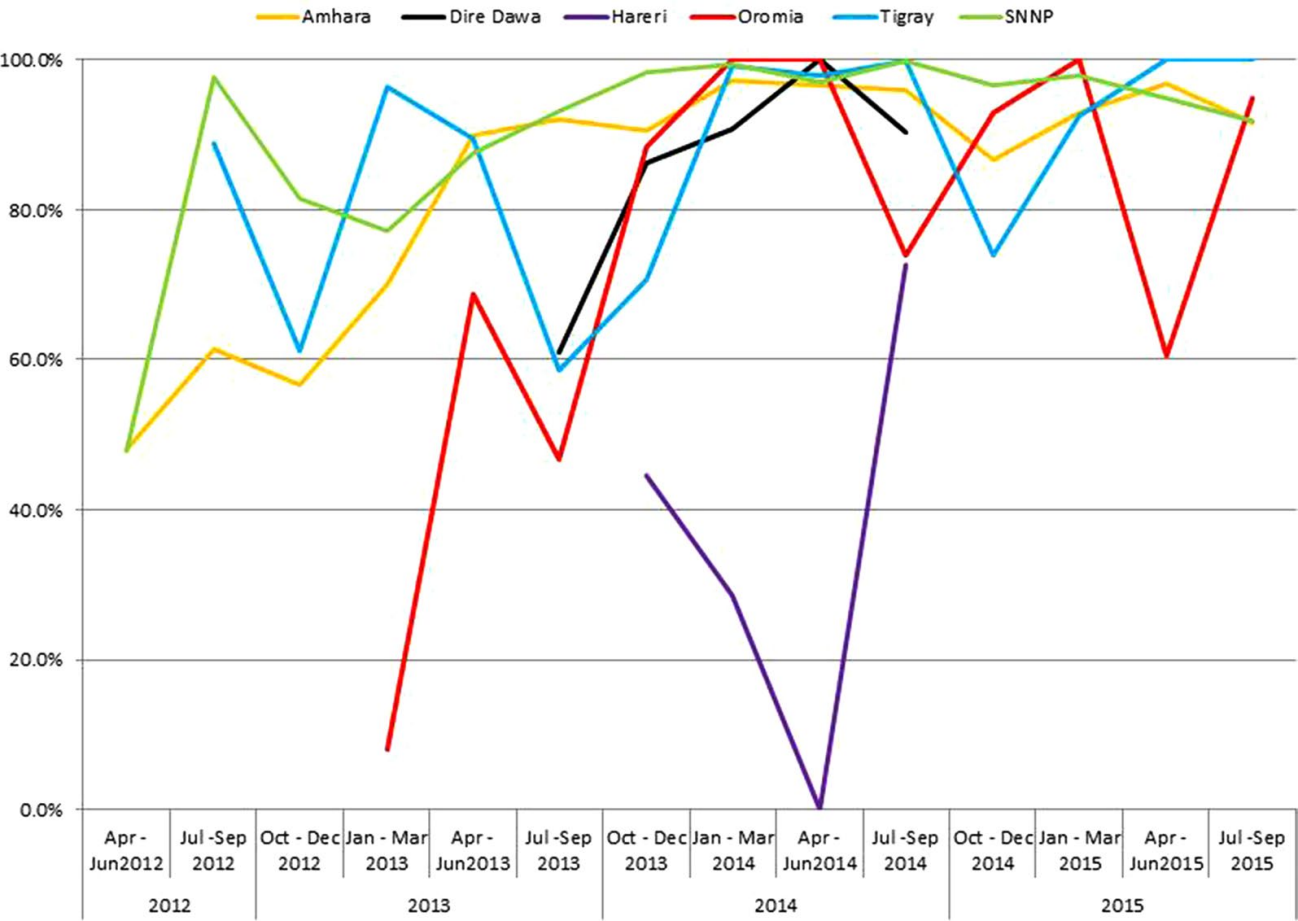
Trends of appropriate treatment using AL (Coartem^®^) in PPP malaria facilities, Ethiopia, 2012–2015. *Line graphs* showing adherence of health care providers to the nationally recommended treatment for *Plasmodium falciparum* malaria, mixed malaria and clinical diagnosis malaria

**Fig. 6:**
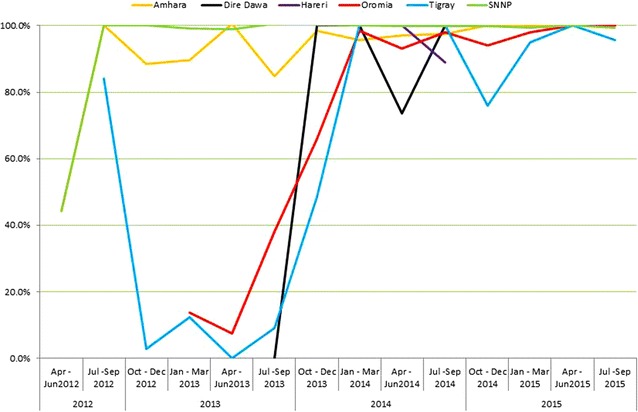
Trends of appropriate treatment using chloroquine (CQ) in PPP malaria facilities, Ethiopia, 2012–2015

In this study, at the time of initiation of PPP for malaria care service (April–June 2012) adherence of health workers to the standards of *P. falciparum* (AL) infection treatment was improved from 47.8 % (2501/5223) to 95.7 % (3417/3567) in the last quarter (July–September 2015) with wide range from 56.0 to 95.1 % achievements for the rest of the quarters (Fig. 5). Similarly, adherence to *P. vivax* treatment (CQ) was 44.1 % (1425/3225) in the first quarter and 98.1 % (3295/3358) in the last quarter with range 76.3–100.0 % of performance was recorded within the study period (Fig. 6), respectively. The temporal changes of improvements in treatment adherence with the national recommended standards were evaluated using with non- parametric statistics McNemar’s test. Computing the changes in improvement of malaria management of the first against the last quarter for AL and CQ was found statistically significant at P < 0.001 (Table 4).

## Discussion

This study determined the magnitude of malaria among self-reported suspected cases in PPP for malaria care facilities from April 2012 through September 2015 in six regional states of Ethiopia. The study describes the prevalence of *Plasmodium* species, service beneficiaries by age category, sex and years. In addition, the study documented the significant improvement in adherence of health care providers with national standards recommended for diagnosis and treatment.

The majority 96.0 % of malaria patients treated in selected 110 PPP for malaria care facilities were parasitological confirmed either using microscopy or RDT. This finding is a little higher than the national estimated 60.0 and 84.1 % confirmed malaria patient treated in private and public health facilities in Ethiopia, respectively [3, 22]. This successful achievement could be related to the effective intervention (technical support, joint supportive supervisions, team trainings, mentorships and access to supplies) made by the RHBs and PHSP.

This study revealed that 60 % of malaria patients surveyed in PPP for malaria facilities were males. But this difference significantly reduced when the age of patients falls in the lower age category. This finding is in line with Yukich et al. [29] and Regassa [30] describe the presence of higher risk of malaria infection among adults and males in Ethiopia. On the other hand adult males might have better economic position and decision power in seeking medical care than females [5]. In Kenya, a result of large national survey documented females are 1.4 times more likely acquire to malaria than males [31].

The majority of service beneficiary were accessed malaria care services from private for profit health facilities. In addition, close to one out of ten patients were served in Private not for the profit; workplace health facilities. This result could be due to the fact that the larger groups of PHSP supported facilities are Private for Profit facilities [24].

The trend of SPR significantly decreases from 39.4 % in the first quarter to 18.4 % in the last quarter (X^2^ = 4.69, P < 0.001). This significant level of result might be attributed to the reduction in burden of malaria across the whole country [32], and might be ascribed to the implemented twelve steps PHSP strategies which ensure the quality of services [24]. The average SPR was 25.1 %. This finding was a little higher than the national estimated slide positivity rate 19.0 % [3]. However, Chala and Pertos [5] for the period ranges from 2001 to 2005 reported overall SPR was 30.9 % in Finchaa Sugar Plantation and Factory site in Ethiopia [5].

In Ethiopia, the two dominant *Plasmodium* species known for causes of malaria infection with annual prevalence were 60–70 % *P. falciparum* and 30–40 % *P. vivax* [2, 4]. Whereas, in this study, almost one half (50.0 %) were found to be for *P. falciparum* and 46.0 % were confirmed P*. vivax*. This research documented a significant difference in proportion of *Plasmodium* species identified using RDT compared to microscopy. The magnitude of *P. falciparum* among patients diagnosed using RDT groups was much higher than patients identified using microscopy. Studies reported wide range of difference in prevalence of *Plasmodium* species for example in North Western Ethiopia 90.0 % *P. falciparum* were documented in 10 years data from Metema Hospital [33], while Regassa (2014) found 64 % *P. falciparum* and 25 % *P. vivax* in SNNP, Arbamich hospital [29].

Figures 4 and 5 depicted the trends of appropriate malaria case management to presumed diagnosis, *P. falciparum* or mixed, and *P. vivax* infections, respectively. The temporal changes in adherence to recommended treatment for presumed diagnosis, *P. falciparum or mixed* infection was improved from 47.8 % in the first quarter to 95.7 % in the last quarter. Similarly, adherence to *P, vivax* infection was improved from 44.1 % in the first quarter and 98.1 % in the last quarter. This finding was much higher than the baseline survey conducted by Argaw (2015) in Ethiopia. However, there are several studies documented improvements in adherence to the standards [10–14, 34–36].

## Limitations

This retrospective descriptive study was made based on collected data for Health Information System. This study unable to determine and analyse other socio demographic characteristics of the clients and other aspects of quality service delivery such as provider client interaction.

## Conclusions

This study documented the chronological changes of adherence of health care providers with the national recommended standards to treat malaria. Scaling up of PPP for malaria care services is recommended through partners and the national malaria prevention control programme.

## Abbreviations

AIDS: acquired immune deficiency syndrome
AL: artemether-lumefantrine
CQ: chloroquine
EFMOH: Ethiopian Federal Ministry of Health
EQA: external quality assurance
FP: family planning
HIV: human immune deficiency virus
IPLS: integrated pharmaceutical logistics management system
IQC: internal quality control
MOU: memorandum of understanding
ODK: open data kit
PHSP: Private Health Sector Programme
PPM: public private mix
PPM- DOTS: public private mix direct observed therapy short course
PPP: public private partnerships
PSP: private sector project
RBM: roll back malaria
RDT: rapid diagnostic test
RHB: regional health bureau
SNNP: Southern Nation Nationalities People
STI: sexually transmitted infections
TB: tuberculosis
USAID: United State Agency for International Development
WHO: World Health Organization
